# Stille Hypoxie bei COVID-19: Eine Fallserie

**DOI:** 10.1159/000521461

**Published:** 2022-01-18

**Authors:** Thomas Fuehner, Isabelle Renger, Tobias Welte, Tobias Freundt, Jens Gottlieb

**Affiliations:** ^a^Klinik für Pneumologie, Intensiv- und Schlafmedizin, KRH Klinikum Siloah, Hannover, Deutschland; ^b^Klinik für Pneumologie und Deutsches Zentrum für Lungenforschung (DZL/BREATH), Medizinische Hochschule Hannover, Hannover, Deutschland

**Keywords:** SARS-CoV-2, Coronavirus-Krankheit 2019, Atemnot, Hypoxämie, Hypokapnie

## Abstract

**Hintergrund:**

Die Coronavirus-Pandemie 2019 (COVID-19) ist eine anhaltende globale Krise, die die weltweiten Gesundheitssysteme herausfordert. Bei vielen Patienten besteht ein Missverhältnis zwischen schwerer Hypoxämie und wenigen Anzeichen von Atemnot (d.h. stille Hypoxämie). Dieses besondere klinische Bild wird häufig angeführt, aber die Daten sind begrenzt.

**Hauptteil:**

Die vorliegende Studie beschreibt das Empfinden von Dyspnoe, das mithilfe der BORG-Skala über einen Zeitraum von 4 Wochen bei Lungenpatienten ermittelt wurde, die in die Notaufnahme aufgenommen und dann in die Lungenabteilung des Siloah-Krankenhauses in Hannover verlegt wurden. Vom 1. Oktober bis zum 1. November 2020 wurden 82 Patienten mit Hypoxämie, definiert mit einem Sauerstoffbedarf zum Erreichen einer Sauerstoffsättigung (SpO<sub>2</sub>) ≥92%, eingeschlossen. Bei 45/82 (55%) Patienten wurde bei der Aufnahme durch PCR SARS-CoV-2 nachgewiesen. Bei den Nicht-COVID-Patienten war die Exazerbation der COPD die Hauptdiagnose (15/37, 41%). Alle Probanden bewerteten ihre wahrgenommene Dyspnoe anhand der modifizierten Borg-CR10-Skala. Die Patienten in der Nicht-COVID-Gruppe litten mehr unter Dyspnoe auf der modifizierten Borg-CR10-Skala (Median 1, IQR: 0–2 vs. Median 5, IQR: 3–6, *p* < 0,001). In der multivariaten Analyse war eine «stille Hypoxämie», definiert durch die Dyspnoe-Borg-CR10-Skala ≥5, unabhängig von COVID-19 und dem Vorhandensein einer schweren Hypokapnie mit einer Odds Ratio von 0,221 (95%-Konfidenzintervall 0,054, 0,907, *p* 0,036) verbunden.

**Schlussfolgerungen:**

Bei Lungenpatienten mit akuter Hypoxämie, die mit einem Sauerstoffbedarf definiert wurden, leiden COVID-19-Patienten im Vergleich zu Nicht-COVID-Patienten weniger unter Dyspnoe. «Stille» Hypoxämie trat bei COVID-19-Patienten häufiger auf.

## Einführung

Bei COVID-19-Patienten wurde eine Hypoxämie mit einer unverhältnismäßig geringen Empfindung von Dyspnoe festgestellt. Dieses Phänomen wird als «stille» oder «glückliche» Hypoxämie bezeichnet [1, 2, 3]. Der zugrunde liegende Mechanismus der stillen Hypoxämie ist nicht vollständig geklärt. Die «stille» Hypoxämie ist nicht auf COVID-19 beschränkt und kann auch bei anderen Atemwegserkrankungen auftreten. Der genaue Anteil der Patienten mit stiller Hypoxämie bei COVID-19 und anderen Atem­wegserkrankungen ist unbekannt. Ziel dieser Studie war es, das Empfinden von Dyspnoe bei hypoxämischen Notfallaufnahmen zu untersuchen.

## Methode

Alle Notfallaufnahmen auf der Beatmungsstation im Siloah-Krankenhaus, Hannover, Deutschland, zwischen dem 1. Oktober und dem 1. November 2020 wurden retrospektiv ausgewertet. Alle Patienten wurden bei der Aufnahme durch Polymerase-Kettenreaktion (polymerase chain reaction, PCR) auf SARS-CoV-2 getestet. Patienten mit Hypoxämie, definiert mit zusätzlichem Sauerstoffbedarf zur Erreichung einer Sauerstoffsättigung (SpO_2_) von ≥92% in den ersten 24 Stunden nach der Aufnahme, wurden eingeschlossen. Die Vitalparameter wurden aufgezeichnet und der nationale Frühwarnwert (NEWS-2) wurde zur Überwachung verwendet [4]. Der Sauerstoffbedarf wurde mittels Kapillarblutgasanalyse titriert [5]. Die Probanden bewerteten ihre wahrgenommene Dyspnoe anhand der modifizierten Borg-CR10-Skala in Lizenz [6], Vertrags-ID 13LX473. Diese Skala umfasst 12 Stufen von 0 bis 10 einschließlich 0,5. Die Zahlen auf dieser Skala beziehen sich auf eine Beschreibung der Atemnot bei Anstrengung. Demografische Daten, chronische Erkrankungen (Krebs, Diabetes mellitus, Bluthochdruck, chronische Atemwegserkrankungen und Adipositas), Anzeichen und Symptome, einschließlich Fieber, Husten, Anosmie, Ageusie, Dyspnoe und Sauerstoffsättigung, sowie die Ergebnisse der Blutgasanalyse wurden analysiert. Die Studie wurde in Übereinstimmung mit den ethischen Richtlinien der Deklaration von Helsinki von 1975 und dem institutionellen Prüfungsausschuss durchgeführt.

### Statistiken

Kontinuierliche Variablen wurden als Median und Quartile von 25 und 75% beschrieben. Kategorische Variablen werden als *n* (%) angegeben. Zum Vergleich der Gruppen wurden der χ^2^-Test oder der exakte Test nach Fisher für kategorische Variablen (falls zutreffend) und der t-Test («Student's-Test») für metrische Variablen nach Prüfung auf Normalverteilung zum Vergleich von Gruppen verwendet. Es wurde ein logistisches Regressionsmodell mit einer Borg-CR10-Skala von ≥5 als kategoriales Ergebnis erstellt. Die einbezogenen Variablen wurden durch einen *p*-Wert von <0,1 in der univariaten Analyse identifiziert.

## Ergebnisse

**Abb. 1:**
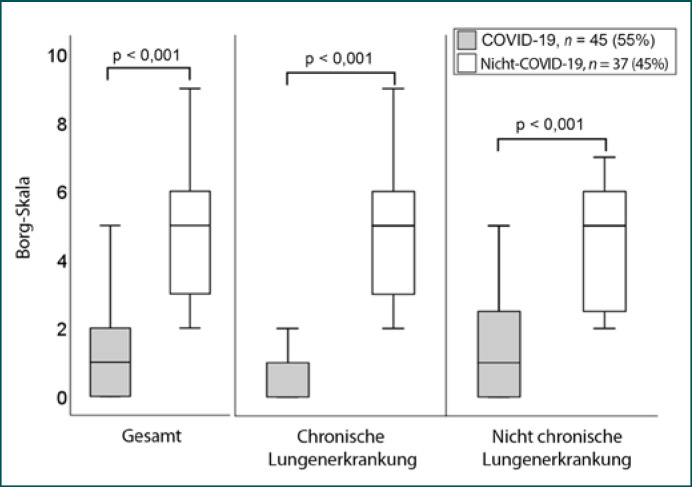
Bewertung der Dyspnoe anhand der Borg-CR10-Skala bei Notfallaufnahmen.

Während des Studienzeitraums wurden 82 Patienten eingeschlossen. Bei 45/82 (55%) Patienten wurde bei der Aufnahme durch PCR SARS-CoV-2 nachgewiesen. Bei 9 von 45 (20%) der COVID-19-Patienten lag eine chronische Lungenerkrankung vor, verglichen mit 28/37 (76%) Patienten aus der Nicht-COVID-Gruppe (Tab 1). Zwei von 37 Patienten (5%) in der Gruppe der Nicht-COVID-Patienten nahmen vor der Krankenhauseinweisung eine häusliche Langzeit-Sauerstoffbehandlung in Anspruch. Von den Nicht-COVID-Patienten litt die Mehrheit (15/37, 41%) an einer akuten Exazerbation der COPD, gefolgt von einer ambulant erworbenen Lungenentzündung (8/37, 22%). In der Nicht-COVID-Gruppe hatten mehr Patienten einen pCO_2_-Wert von ≥45 mm Hg. Kein COVID-19-Patient war hyperkapnisch. Die mit der Borg-CR10-Skala bewertete Dyspnoe war in der COVID-19-Gruppe geringer, auch bei Personen mit chronischen Lungenerkrankungen (Abb 1). Alle bis auf einen Nicht-COVID-19-Patienten (98%) hatten einen Wert von <5 auf der Borg-CR10-Skala. Es gab keinen Unterschied in der Dauer des Krankenhausaufenthalts (Median 10 gegenüber 8 Tage, *p* = 0,189), der Aufnahme auf die Intensivstation (7/45 gegenüber 8/37, *p* = 0,48), der invasiven Beatmung (2/45 gegenüber 2/37, *p* = 1,0) oder der Sterblichkeit (6/45 gegenüber 2/37, *p* = 0,28) zwischen COVID- und Nicht-COVID-Patienten. In der multivariaten Analyse war eine stille Hypoxämie, definiert durch die Dyspnoe-Borg-CR10-Skala ≥5, unabhängig von COVID-19 und dem Vorhandensein einer schweren Hypokapnie mit einer Odds Ratio von 0,221 (95%-Konfidenzintervall 0,054, 0,907, *p* 0,036) verbunden (Tab 2).

## Diskussion

Nach unserem Kenntnisstand ist dies die erste Fallserie, die das Empfinden von Dyspnoe bei COVID-19-Patienten im Vergleich zu Patienten mit anderen Atemwegserkrankungen systematisch analysiert. Patienten mit COVID-19 hatten bei ähnlichem Sauerstoffpartialdruck ein höheres Dyspnoe-Empfinden, das anhand der BORG-Skala bewertet wurde.

Dyspnoe als Kurzatmigkeit wird bei ambulant erworbenen Lungenentzündungen und COPD-Exazerbationen in 75% bzw. 78% häufig berichtet [7, 8]. Docherty et al. [9] berichteten in einer großen Beobachtungskohorte von 20 133 Krankenhauseinweisungen aufgrund von COVID-19 über Kurzatmigkeit bei 71%. Im Gegensatz dazu und ähnlich wie bei unseren Ergebnissen klagten bei 1712 stationären COVID-19-Patienten (zwei Drittel mit Lungenentzündung) 65% bei der Aufnahme nicht über Kurzatmigkeit [10]. Leider wurde in diesen Veröffentlichungen keine genaue Definition von Atemnot verwendet oder die Dyspnoe nicht objektiv bewertet.

Die Hypoxämie bei COVID-19 wird durch intrapulmonale Shunts, den Verlust der Lungenperfusionsregulation, intravasale Mikrothromben, eine beeinträchtigte Diffusionskapazität und den Erhalt der Lungenmechanik verursacht [11, 12, 13, 14]. Interessanterweise unterscheiden sich die Kompensationsmechanismen der Atmung je nach der zugrunde liegenden Lungenpathologie erheblich. Patienten mit COVID-19 und Hypoxämie neigen dazu, dies durch Hyperventilation zu kompensieren und sind in der Regel hypokapnisch, wie in unserer Studie bestätigt wurde. Dieser Kompensationsmechanismus der Atemwege scheint ähnlich zu sein wie bei einer ambulant erworbenen Lungenentzündung oder einer interstitiellen Lungenerkrankung. Bei beiden Entitäten ist die Dyspnoe ein Hauptsymptom. Kürzlich wurde angenommen, dass eine hypokapnische Hypoxämie ohne Dyspnoe durch einen intrapulmonalen Rechts-Links-Shunt bei COVID-19 verursacht wird [14, 15].

Hypoxämie korreliert nur bedingt mit dem Gefühl der Atemnot, während Dyspnoe eher mit Hyperkapnie korreliert [16, 17]. Die «stille Hypoxämie» bei COVID-19 kann durch Veränderungen im Atemkontrollsystem verursacht werden. Angiotensin-Converting-Enzym-2-Rezeptoren sind sowohl in der Nasenschleimhaut als auch in den Karotiskörpern weit verbreitet, wo sich die Sauerstoff-Chemorezeptoren zur Regulierung der Atmung befinden. Ein weiteres Beispiel für neuronale Störungen bei COVID-19 ist die Anosmie, die bei einem Drittel der Patienten mit COVID-19 auftritt [10, 18]. Die stille Hypoxämie bei COVID-19 sollte weiter untersucht werden, um ihren Mechanismus zu klären.

Die Fallserie weist potenzielle Beschränkungen auf, die sich aus dem monozentrischen Studiendesign mit einer geringen Teilnehmerzahl und dem daraus resultierenden Mangel an Untergruppenanalysen ergeben. Virusvarianten wurden bis zu diesem Zeitpunkt in der klinischen Routine nicht untersucht. Darüber hi­naus ist die Borg-CR10-Skala ein Instrument zur Messung der Dyspnoe bei körperlicher Aktivität. Die Skala ist nicht zur Bewertung der Dyspnoe bei Patienten mit akuter Hypoxämie in Ruhe geeignet.

## Fazit

Eine stille Hypoxämie tritt bei COVID-19-Patienten häufiger auf als bei Lungenpatienten mit akuter Hypoxämie. Eine weitere Bewertung ihrer Einzigartigkeit und ihrer pathophysiologischen Mechanismen ist erforderlich.

## Danksagung

Die Autoren danken Christina Valtin, Abteilung für Atemwegsmedizin und Deutsches Zentrum für Lungenforschung (DZL/BREATH), Medizinische Hochschule Hannover, Hannover, Deutschland.

## Ethikerklärung

Die Daten aus der klinischen Routine wurden retrospektiv ausgewertet. Die Studie wurde in Übereinstimmung mit dem Institutional Review Board, KRH Klinikum Region Hannover, durchgeführt. Alle Verfahren, einschließlich der Einverständniserklärung, wurden in Übereinstimmung mit den ethischen Normen des zuständigen Ausschusses für Experimente am Menschen (auf institutioneller und nationaler Ebene) und der Helsinki-Erklärung aus dem Jahr 1975 in der im Jahr 2000 überarbeiteten Fassung durchgeführt.

## Interessenskonflikte

Die Autoren erklären, dass keine Interessenskonflikte bestehen.

## Finanzierungsquellen

Es wurden keine Fördermittel erhalten.

## Beiträge der Verfasser

T.F. war an der Konzeption der Studie beteiligt, führte die statistische Analyse durch, konzipierte die Studie, beteiligte sich an deren Gestaltung und Koordination und half bei der Abfassung des Manuskripts. I.R. war an der Planung der Studie beteiligt, konzipierte die Studie, beteiligte sich an ihrer Gestaltung und Koordinierung und half bei der Abfassung des Manuskripts. T.W. konzipierte die Studie, beteiligte sich an ihrer Planung und Koordination und half bei der Abfassung des Manuskripts. T.F. war an der Planung der Studie beteiligt, konzipierte sie und half bei der Abfassung des Manuskripts. J.G. war an der Konzeption der Studie beteiligt, führte die statistische Analyse durch, konzipierte die Studie, beteiligte sich an deren Gestaltung und Koordination und half bei der Abfassung des Manuskripts. Alle Verfasser haben das endgültige Manuskript gelesen und genehmigt.

## Erklärung zur Datenverfügbarkeit

Die im Manuskript beschriebene Software, Datenbanken und Anwendung/Tool stehen den Gutachtern zum Testen zur Verfügung.

## Lizenzangabe

Thomas Fuehner, Isabelle Renger, Tobias Welte, Tobias Freundt, Jens Gottlieb: Silent Hypoxia in COVID-19: A Case Series. Respiration. 2021:1–5 (DOI: 10.1159/000520083). ^©^2021 Die Autoren (Übersetzung), lizensiert unter CC BY 4.0 (https://creativecommons.org/licenses/by/4.0/deed.de).

## Figures and Tables

**Tab. 1:** Klinische Merkmale

	Gesamt	COVID-19	Nicht-COVID-19	p-Wert
*N* (%)	82 (100)	45 (55)	37 (45)	
Geschlecht weiblich, *n* (%)	26 (32)	15 (33)	11 (30)	0,727
Alter, Median (25, 75 Perzentil), Jahre	74 (63, 80)	69 (57, 78)	76 (68, 82)	0,032
Chronische Erkrankung der Luftwege, *n* (%)	37 (45)	9 (20)	28 (76)	<0,001
COPD	17 (21)	2 (4)	15 (41)	<0,001
Asthma	6 (7)	3 (7)	3 (8)	1,000
Lungenkrebs	9 (11)	4 (9)	5 (14)	0,501
ILD	6 (7)	-	6 (16)	0,006
Hauptdiagnose, *n* (%)				
COVID-19	45 (55)	45 (100)		
Malignität	6 (7)		6 (16)	
Ambulant erworbene Pneumonie	8 (10)		8 (22)	
AECOPD	13 (16)		13 (35)	<0,001
AE-ILD	4 (5)		4 (11)	
AE Asthma	2 (2)		2 (5)	
Kongestive Herzinsuffizienz	3 (4)		3 (8)	
Sauerstoffflussrate, Median (25, 75 Perzentil), l/min	2 (2, 4)	2 (2, 4)	2 (2, 4)	0,996
Sauerstoffsättigung, Median (25, 75 Perzentil), %	92 (92, 94)	92 (92, 94)	92 (92, 94)	0,195
Atemfrequenz, Median (25, 75 Perzentil)	21 (20, 23)	21 (20, 22)	22 (20, 24)	0,664
Tachypnoe, Atemfrequenz >20/min, *n* (%)	46 (56)	24 (53)	22 (60)	0,578
Temperatur, Median (25, 75 Perzentil), ° C	37,2 (36,8, 37,8)	37,1 (36,8, 37,7)	37,4 (37, 38)	0,126
Systolischer Blutdruck, Median (25, 75 Perzentil), mm Hg	130 (120, 140)	130 (120, 140)	130 (120, 145)	0,196
Herzfrequenz, Median (25, 75 Perzentil), bpm	80 (72, 86)	78 (69, 85)	81 (78, 89)	0,014
Frühwarnwert, Median (25, 75 Perzentil)	6 (4, 6)	5 (3, 6)	6 (4,7)	0,020
pH, Median (25, 75 Perzentil)	7,43 (7,40, 7,44)	7,44 (7,40, 7,45)	7,43 (7,35, 7,44)	0,121
BNP, Median (25, 75 Perzentil)	256 (100 1298)	193 (82, 352)	2200 (100, 6,750)	0,022
pO_2_, Median (25, 75 Perzentil), mm Hg	64 (61, 66)	64 (61, 66)	63 (60, 70)	0,779
pCO_2_, Median (25, 75 Perzentil), mm Hg	33 (31, 39)	32 (31, 36)	36 (31, 50)	0,003
pCO_2_< 35 mm Hg, *n* (%)	48 (59)	32 (71)	16 (43)	<0,001
pCO_2_≥ 45 mm Hg, *n* (%)	15 (18)	-	15 (41)	<0,001
AaDo2, Median (25, 75 Perzentil), mm Hg	**109 (98, 140)**	**107 (100, 161)**	**110 (95, 136)**	**0,144**
CRP, Median (25, 75 Perzentil), mg/dl	74 (54, 92)	75 (55, 95)	69 (52, 91)	0,699
D-Dimer, Median (25, 75 Perzentil)	1,00 (0,86, 2,01)	1,00 (0,89, 1,80)	0,50 (0,50, 7,20)	0,697
Infiltrate	**79 (96)**	**43 (96)**	**36 (97)**	**1,000**
Spezifische Therapie (mehrere Elemente möglich), *n* (%)				
Steroide	48 (59)	38 (84)	10 (27)	<0,001
Antibiotikatherapie	33 (40)	-	33 (89)	<0,001
Remdesivir	6 (7)	6 (13)	-	0,030

COVID-19, Coronavirus-Krankheit 2019; COPD, chronisch obstruktive Lungenerkrankung; ILD, interstitielle Lungenerkrankung; AE, akute Exazerbation; mm Hg, Millimeter Quecksilbersäule; bpm, Schläge pro Minute; BNP, natriuretisches Peptid des Gehirns; pO_2_, Sauerstoffpartialdruck; pCO_2_, Partialdruck von Kohlendioxid.

**Tab. 2:** Multivariate Analyse

Kovariate Borg ≥5	*N*	Dyspnoe (n = 22) (28 %)	Keine Dyspnoe (n = 56) (72 %)	Chancen-Verhältnis	95%-Konfidenz-intervall	p-VVert
COVID-19, *n* (%)	78					
Nein	36 (46)	21 (58)	15 (42)	(Ref)	(Ref)	(Ref)
Ja	42 (54)	1 (2)	41 (98)	0,007	0,000–0,199	0,004
pO_2_, Median (25, 75 Perzentil), mm Hg	78	62 (58, 65)	65 (62, 66)	0,845	0,671–1,063	0,150
pCO_2_, Median (25, 75 Perzentil), mm Hg	78	36 (31, 52)	33 (31, 36)	0,816	0,627–1,061	0,129
Chronische Lungenkrankheit, *n* (%)	78					
Nein	41 (53)	5 (12)	36 (88)	(Ref)	(Ref)	(Ref)
Ja	37 (47)	17 (46)	20 (54)	0,705	0,038–13,206	0,815
pH <7,35, *n* (%)	78					
Nein	68 (87)	15 (22)	53 (78)	(Ref)	(Ref)	(Ref)
Ja	10 (13)	7 (70)	3 (30)	95,816	1,744–5,263–453	0,026
NIV, *n* (%)	78					
Nein	72 (92)	18 (25)	54 (75)	(Ref)	(Ref)	(Ref)
Ja	6 (8)	4 (67)	2 (33)	4,305	0,280–66,176	0,295
Lungenentzündung, *n* (%)	78					
Nein	25 (32)	16 (64)	9 (36)	(Ref)	(Ref)	(Ref)
Ja	53 (68)	6 (11)	47 (89)	1,391	0,060–32,408	0,837
Atemfrequenz, Median (25, 75 Perzentil)	78	24 (21, 28)	21 (20, 22)	1,271	0,968–1,669	0,084
Herzfrequenz, Median (25, 75 Perzentil)	78	86 (78, 90)	80 (71, 85)	1,086	0,983–1,199	0,105
Frühwarnwert, Median (25, 75 Perzentil)	78	6 (5, 7)	5 (3, 6)	1,060	0,448–2,507	0,894
AaDo2, Median (25, 75 Perzentil)	78	124 (110, 141)	102 (97, 150)	0,936	0,785–1,116	0,461
O_2_-Flussrate, Median (25, 75 Perzentil)	78	3 (2, 4)	2 (2, 4)	6,214	0,052–737,097	0,453

Ref, Referenzkategorie; COVID-19, Coronavirus-Krankheit 2019; mm Hg, Millimeter Quecksilbersäule; pO_2_, Sauerstoffpartialdruck; pCO_2_, Partialdruck von Kohlendioxid.
